# Fermentation Performance of Carob Flour, Proso Millet Flour and Bran for Gluten-Free Flat-Bread

**DOI:** 10.3390/foods13213458

**Published:** 2024-10-29

**Authors:** Bojana Voučko, Nikolina Čukelj Mustač, Ljiljana Nanjara, Saša Drakula, Tomislava Grgić, Duška Ćurić, Dubravka Novotni

**Affiliations:** 1 Department of Food Engineering, Faculty of Food Technology and Biotechnology, University of Zagreb, Pierottijeva 6, 10000 Zagreb, Croatia; 2 Department of Food Technology, University of Applied Sciences “Marko Marulić”, Kralja Petra Krešimira IV 30, 22300 Knin, Croatia

**Keywords:** *Kluyveromyces marxianus*, *Limosilactobacillus fermentum*, optimization, sourdough, starter culture

## Abstract

Sourdough fermentation is rarely used for gluten-free flatbread (GFFB), a product that is challenging to produce, especially when using high-fiber ingredients that bring nutritional benefits but lead to physical deterioration. The aim of this study was therefore to evaluate the fermentation performance of carob flour (CSPF), proso millet flour (PMF), and proso millet bran (PMB) individually and in combination with *Limosilactobacillus fermentum* and *Kluyveromyces marxianus* (LF + KM) and to compare the performance of LF + KM with a commercial starter (LIVENDO^®^ LV1). A mixture design (n = 13) was used to evaluate the fermentation performance of LF + KM (total titratable acidity (TTA); lactobacilli and yeast growth; acetic and lactic acid, fructose, glucose, and saccharose content) at 35 °C for 16 h. The comparison of LF + KM with LV1 fermentation was based on the acidity rate, fermentation quotient, TTA, and finally by determining the physical properties (texture, shape, color) of a rice–corn GFFB in which 10% of flour was supplemented with the sourdoughs. PMB promoted the growth of lactobacilli and the production of organic acids, especially in combination of CSPF and PMF. The optimum flour ratio was 2.4:1:1.2 (PMB:PMF:CSPF). LF + KM shortened the sourdough fermentation time by 2.5 times compared to LV1. The use of LF + KM sourdough reduced the hardness (32%) and chewiness (28%) of the GFFB, while the volume (35%) was increased compared to LV1 sourdough. This study shows the potential of using local alternative flours in sourdough fermentation for the production of GFFB.

## 1. Introduction

“Flat” breads are popular due to their simplicity and are characterized by being relatively thin, which makes them suitable for the use of pseudocereals or legumes [1]. Despite their popularity, there is still much room for technical improvement, especially in nutritionally and texturally poorer gluten-free varieties of flatbread. Problems that are typical for gluten-free baked goods, e.g., that the recipes are based on starch, and mostly produced of rice or corn flour, also exist for flatbread. This results in a lack of variety in nutrients, and lower protein variety and fiber content than gluten-containing varieties [2]. Despite the growing market for gluten-free products, it is still a challenge to produce a gluten-free bread that fulfils both the nutritional content and texture properties [3]. Improvement in acceptability of gluten-free bread among consumers can be achieved through formulation and process adjustments, where nowadays, there is preference for natural improvers. For example, alternative flours and pseudocereals have the potential to improve nutritional content and sensory properties when fermented and incorporated into gluten-free bread recipes [4]. An alternative raw material traditionally used in the Mediterranean region is carob. Due to its high mineral and fiber content [5], the use of carob improves the nutritional value of bread [6]. In addition, bean flours such as soybean, chickpea, and carob can improve viscoelastic properties and are therefore interesting as functional ingredients in gluten-free bread recipes [7]. In contrast to its application in gluten-free breads, the potential of adding carob fiber and carob flour to wheat bread has been confirmed [8,9,10]. The demand for gluten-free products made from millet is also increasing due to its resilience in cultivation, nutritional value, and potential health benefits [11]. Different millet varieties include pearl, finger, proso, kodo, and foxtail millet, all of which have a specific nutritional profile. When properly formulated and processed, proso millet flour can be a promising ingredient for the production of gluten-free bread products with improved sensory and nutritional properties due to its protein content. [12]. To improve their nutritional value and maintain their sensory properties, bakery products can be enriched with up to 15% of bran [13]. A nutrient-dense byproduct of millet processing, millet bran has the potential to improve gluten-free bread recipes, as shown in the research by Čukelj Mustač et al. [14], in which the addition of millet bran to gluten-free bread led to a significant increase in phenolic content and dietary fiber. However, sensory and rheological qualities are adversely affected by the addition of bran to classic recipes [13]. Sourdough fermentation represents an environmentally friendly biotechnological process that enables fortification with bran [15]. Excessive addition of sourdough can have a negative effect on bread volume and texture [16]. Researchers have successfully produced gluten-free sourdough products with millet, brown rice, and commercial gluten-free flour blends, with varying results in terms of texture and sensory properties [17]. Proso millet is the least used in improvement of gluten-free bread, while its use in sourdough for gluten-free bread has still not been published. Additionally, while increased antioxidant activity and dietary fiber content have been demonstrated when fermenting carob flour into sourdough and adding it to wheat bread [6], the results of using fermented carob flour in gluten-free production have not been published. To maximize the potential of sourdough fermentation, process optimization and careful selection of microbial species are required [18]. Choosing the appropriate starter culture is crucial, since different strains of lactic acid bacteria can improve bread quality in different ways [19] and enable quick adaptation, strong acidification, and an overall positive impact on nutrition and technology [18]. The efficiency of commercial starters varies with the substrate and the circumstances of fermentation [20]. While the microbial composition, functional properties, and organoleptic qualities of rye and wheat sourdoughs are well researched, little is known about gluten-free sourdoughs. *Limosilactobacillus fermentum* is an example of the natural thermophilic microbiota [18] and has been isolated from a variety of gluten-free sourdoughs [21]. *Kluyveromyces marxianus* is a promising yeast for use in a variety of food and biotechnological applications. Recently, it has been successfully used in rye and wheat sourdough for the production of bread and snacks, where it led to a reduction in fructan content [22,23]. In white kidney bean sourdough, *K. marxianus* in combination with *Lactobacillus plantarum* or *Pediococcus pentosaceus* reduced antinutritional factors and improved dough microstructure [24]. The rapid growth rate and thermotolerance of *Kluyveromyces marxianus* [25] show its potential for use in gluten-free sourdough. The combination of *Limosilactobacillus fermentum* with *Kluyveromyces marxianus* has not yet been published in the production of gluten-free sourdough. Therefore, this research aimed at an optimization of the fermentation performance of local, traditional, and alternative flours with novel combinations of starter cultures. In addition, the optimized fermentation of the selected starter cultures was compared with a commercial one by investigating the effects of the different sourdoughs on the physical properties of the gluten-free flatbread.

## 2. Materials and Methods

### 2.1. Ingredients

Whole-grain rice flour (Nutrigold, Bulgaria) contained 6% protein, 11.6% moisture, 0.8% ash, and 0.5% fat; white corn flour (Danijel Sinković, a family-run farm, Bedekovčina, Croatia) contained 7% protein, 12.4% moisture, 1.2% minerals, and 3.5% fat; proso millet flour (PMF) (BEZGLUTEN, Posądza, Poland) contained 11% protein, 11.3% moisture, 1.4% minerals, 3.2% fat; proso millet bran (PMB) (Mlinopek l.c., Murska Sobota, Slovenia) contained 11% protein, 10.9% moisture, 5.6% minerals, and 7.45% fat; carob seed and pod flour (CSPF), (Šafram Inc., Zagreb, Croatia) contained 5% protein, 14% moisture, 2.8% minerals, and 0.53% fat; while carob seed flour (CSF) (a family-run farm, Šipan, Croatia) contained 7.5% protein, 8.9% moisture, 1.68% minerals, and 0.75% fat. The chemical composition of raw materials was according to the manufacturer’s specifications. Other raw materials used to prepare sourdough and bread were tap water, salt (Solana Pag, Pag, Croatia), sugar (Viro, Virovitica, Croatia), sodium bicarbonate (Franck Inc., Zagreb, Croatia), sunflower oil (Zvijezda Ltd., Zagreb, Croatia), dry instant yeast (Lesaffre Adriatic, Prigorje Brdovečko, Croatia), and spray oil for baking (Trennaktiv PR 100, DÜBÖR Groneweg GmbH, Bad Salzuflen, Germany). A commercial freeze-dried starter LIVENDO^®^ LV1 (LV1) (Lesaffre, Cérences, France). *Limosilactobacillus fermentum* (LF) (DSM 20052) was provided by Deutsche Sammlung von Mikroorganismen and Zellkulturen (DSMZ, Braunschweig, Germany), while *Kluyveromyces marxianus* (KM) (NBRC 1777) was donated by the Laboratory for Biochemical Engineering, Industrial Microbiology and Malting and Brewing Technology, University of Zagreb Faculty of Food Technology and Biotechnology (Zagreb, Croatia).

### 2.2. Experiment Design

The experiment was designed using Design Expert v.11 software (StatEase, Minneapolis, MN, USA). An L-optimal mixture design (Table 1) included three variables (amount of PMF, PMB, and CSPF) tested at 6 levels (0; 16.7; 33.3; 50; 66.7; 100%), with a total of 13 experiments and repetitions at 3 points. The responses assessed to evaluate the interactions of PMF, PMB, and CSPF (Table 1, *p* values) were pH at the end of fermentation, lactic acid bacteria (LAB) cell and yeast count, total titratable acidity (TTA), and L-lactic acid (L-LA) and acetic acid (AA) content. The parameters with a *p* value < 0.05 were considered significant and used in the optimization, in order to determine the share of each flour that leads to maximum desirability of sourdough. L-LA was assigned maximum importance (5) while TTA and AA were assigned equal medium (3) importance, and maximum desirability was achieved by maximizing L-LA and TTA, while minimizing AA, while pH at the end of fermentation, yeast, and LAB count were marked in range.

### 2.3. Sourdough Fermentation

A combination of LF and KM (LF + KM) was used to prepare sourdough. LF was propagated in MRS broth (de Man, Rogosa and Sharpe; Biolife, Bolzano, Italy, according to the manufacturer’s instructions) while the broth medium for the propagation of KM contained yeast extract, peptone, and glucose (1, 2, and 2% *w*/*v*, respectively). Both pre-cultures were incubated at 37 °C. After centrifugation, the cultures were dissolved in sterile water. The detailed procedure for culture propagation is described in Drakula et al. (2021) [19].

Sourdoughs were prepared with 100 g of floury material composed of PMF, PMB, and CSPF according to the experimental design (Table 1) and the optimal solution ratio of 2.4:1:1.2, with a dough yield (DY) of 400, in a 1:3 flour-to-water ratio, and an inoculum of *Limosilactobacillus fermentum* (LF; 10^7^ CFU/g) in combination with *Kluyveromyces marxianus* (KM; 10^5^ CFU/g). The dough was fermented in sealed jars in a thermostat (INB 500, Memmert, Schwabach, Germany) at 35 °C for 16 h. The ratio of flours determined in optimization was used for preparation of dough for gluten-free single-layered bread (GFFB), using the LF + KM combination, as well as the commercial starter Livendo^®^LV1 (LV1) containing bacteria of the *Lactobacillus* spp. and yeast. When sourdough was prepared to use in GFFB preparation, it was fermented in the same conditions, but until pH value 4.1, which was 16 h with LV1, and 6.5 h with LF + KM. The pH of 4.1 was chosen as it is the median value of the most common range between 3.4 and 4.9 [21].

### 2.4. Measurement of pH Value and Total Titratable Acidity (TTA) and Viable Cell Counts in Sourdough

The pH electrode was immersed in the sourdough and the pH value was recorded every 10 min during sourdough fermentation, using a PH-230SD pH meter and data logger (Lutron Electronic Enterprise Co., Ltd., Taipei City, Taiwan). Acidification data of three replicated sourdough fermentations were modelled according to the Gompertz equation as modified by Zwietering et al. [26]. The TTA was determined by the method of Lefebvre et al. [27] until the pH of 8.5. At the end of fermentation, the number of viable cells in sourdough was determined according to the ISO 7954:2002 [28] for yeasts and ISO 15214:1998 [29] for LAB. Three decimal dilutions were analyzed in two replicates. The results are expressed as colony-forming units (CFU) per g of sourdough.

### 2.5. HPLC Determination of Organic Acids and Fermentable Sugars in Sourdough

Samples were prepared for organic acid and sugar contents determination following the method by Drakula et al. [19]. The following chemicals were used: potassium hexacyanoferrate (II) trihydrate (≥99%, Kemika, Croatia), zinc sulfate heptahydrate (≥99.5%, Kemika, Croatia), L(+)-lactic acid (≥98%, Sigma-Aldrich, St. Louis, MO, USA), acetic acid (≥98%, Macron Fine Chemicals, Center Valley, PA, USA), sulfuric acid (96%, Carlo Erba, Val-de-Reuil, France), acetonitrile (≥99.9% Sigma Aldrich, USA), saccharose (≥99.5% Sigma Aldrich, USA), D-(+)-glucose (≥99.5% Sigma Aldrich, USA), and fructose (≥99.0% Sigma Aldrich, USA). Determination of L-LA and AA was performed using HPLC Agilent 1200 Series (Agilent Technologies, Santa Clara, CA, USA) with a diode array detector (DAD; G1315D, Agilent) and a MetaCarb 67H column, 300 mm × 6.5 mm (Agilent Technologies, Santa Clara, CA, USA). Equations for the calibration curves were y = 1424.5x + 120.18 (R^2^ = 0.9997) for L-LA, and y = 892.06x + 62.775 (R^2^ = 0.9971) for AA. Sugar content determination was performed using an Agilent 1260 Series (Agilent Technologies) with an RI detector, and a Shodex Asah NH2P-50 4E column (Showa Denko, Tokyo, Japan). Equations for the calibration curves were y = 91.64x + 3782.36 (R^2^ = 0.9999) for saccharose, y = 92.15x − 665.70 (R^2^ = 0.9999) for glucose, and y = 88.95x − 1572.29 (R^2^ = 0.9999) for fructose. Fermentation quotient was calculated as the molar ratio of L-LA to AA. In all equations for the calibration curves y = ax + b, the “x” value stands for the concentration, “a” represents the slope, the “y” value stands for the peak area, and the “b” represents the y-intercept.

### 2.6. Preparation of Bread

PMF, PMB, and CSPF were fermented to sourdough as described in Section 2.5 and added to whole-grain rice flour (45%) and white-corn flour (45%) to make bread dough. The amount of sourdough that was added to the mixture was adjusted so that the weight of flours from sourdough equaled 10% of the total weight of flour. Beforehand, white corn flour was scalded with 110% of boiling hot tap water (flour basis) in a plastic container, mixed for 3 min (Robert Bosch GmbH, Gerlingen, Germany) and cooled to room temperature for 10 min (to reach 42 °C), wrapped in wrapping foil, and kept in a refrigerator at 4–8 °C until baking (24 h). Sugar (5.1%), dry yeast (1.3%), sunflower oil (1%), Na bicarbonate (0.66%), salt (1.5%), and tap water (115%) were incorporated in all recipes at a constant amount (on flour basis). Yeast was pre-fermented in water for 10 min, at 35 °C, at 85% humidity (WIESHEU Wolfen GmbH, Bitterfeld-Wolfen, Germany) before addition to dough. Dough was mixed (Robert Bosch GmbH, Gerlingen, Germany) at medium speed in two consecutive 20 min steps. At the beginning of the first step of mixing, whole rice flour, white corn dough, sourdough, and water were added while yeast was added 10 min after. Remaining ingredients were added to the dough at the beginning of the second phase of mixing, except salt, which was added two minutes before the end of mixing. Liquid dough (100 g) was then transferred to aluminum molds (d = 13 cm) and fermented at 35 °C, at 85% humidity. According to preliminary research, optimal bread fermentation for the bread prepared with sourdough made with CSPF and LF + KM (CSPF_LF + KM) was set for 30 min since longer fermentation time led to a visual drop in volume and porosity. Since the dough prepared with sourdough made with CSPF and LV1 (CSPF_LV1) of CSF and LV1 (CSF_LV1) kept developing throughout fermentation, its bread fermentation was set for one hour. Breads were backed in a deck oven (Ramalhos S.A., Águeda, Portugal) at 280 °C for 3.5 min, flipped, and baked for additional 2.5 min in the mold. Bread was cooled at room temperature for one hour on a porous pan.

### 2.7. Evaluation of Physical Properties of Bread

Bread volume according to AACC Method 10−05.01 [30] and bread weight were determined one hour after baking. Volume/mass ratio was calculated in order to determine the specific volume. The width and height of the bread were measured with a caliper (at min. 3 points) and a spread ratio was calculated as their ratio. Baking loss was calculated according to the following equation:

Baking loss = ((m1 − m2))/m1 × 100

where m1 is the mass of bread dough prior to proofing and m2 is the mass of bread one hour post-baking. Texture profile of bread was determined according to Armero and Collar [31] one hour after baking using a TA1 texture analyzer (Ametek Llyod Instruments Ltd., Bognor Regis, UK), fitted with an aluminum probe 55 mm in diameter and coupled with Nexygen PLUS 3 Software. The sample consisted of two 35 mm round diameter pieces of bread placed on top of each other. Texture profile analysis (TPA) was carried out at test speed 2 mm/s, trigger force 5N, strain 50%, time between two compressions 30 s (in minimum six replicates). The L* a* b* color system was used for determining bread crust color using a Konica Minolta CM-700d colorimeter (Minolta, Tokyo, Japan) with a 7 mm slit cover. The browning index (BI) for each snack after baking was calculated using the following equation:

BI = 100 × ((((a* + 1.75 × L*)/(5.645 × L* + a* − 3.012 × b*)) − 0.31)/0.17)

### 2.8. Statistical Analysis

Response surface plots were produced with the use of Design expert 7.1.3. software. Factorial analysis of variance (ANOVA), post-hoc Tukey HSD test (α = 0.05), correlation analysis, and acidification kinetics modelling were performed using Statistica 14.1.0 (TIBCO Software Inc., Palo Alto, CA, USA).

## 3. Results and Discussion

### 3.1. Optimization of Fermentation of PMF, PMB, and CF

The LAB cell count at the end of all fermentations (Table 2) was in line with the literature, data referring to an expected LAB cell count of log 3 to log 9 CFU/g and a yeast cell count of log 5 to log 7 CFU/g [21]. The lowest CFU/g of LAB as well as the minimum ratio of LAB to yeast (CFU/g) of 1:1 (Table 2) was found in the fermentation of millet flour only (Table 1, run 1). This fermentation also resulted in the highest number of yeast CFU/g and the lowest sugar content at the end of fermentation, indicating that PMF alone is not an optimal medium for sourdough fermentation with LF + KM. The ratio of LAB/yeast (CFU/g) was also low (10:1) in the fermentation where only CSPF was used as the medium (run 3). Similar results to PMF for yeast count and sugar content were also found in the fermentation of only PMB (run 11), but in the case of bran fermentation, the ratio of LAB to yeast at the end of fermentation was 100:1. Optimal sourdough performance is achieved with a ratio of 10:1 to 100:1 as long as the cell densities of lactic acid bacteria and yeasts are not below log 8.0 and 6.0 CFU/g, respectively [21]. Nevertheless, the ratio between LAB and yeasts can vary greatly, from 10.000:1 to 10:1 [32]. The highest LAB count and the highest LAB/yeast ratio were obtained when all three flours were fermented in equal amounts (run 9), indicating an excellent complementary effect of the three flours. The addition of flours rich in fructo-oligosaccharides, such as carob, has a positive effect on LAB growth [33], while the addition of minerals, such as PMB, can promote the growth of LAB in sourdough [21]. A higher LAB count was also found in the fermentation that contained all three flours but had the highest bran content (run 6). This fermentation had a 1000:1 ratio of LAB to yeast cells and had a significantly lower amount of fructose and glucose that was not completely consumed at the end of fermentation, confirming the satisfactory effect of using PMB in combination with CSPF. Since this fermentation has an overall dominance of lactobacilli, while yeasts predominantly ferment glucose [34], the inhibition of yeast growth resulted in remaining sugars at the end of fermentation. The remaining sugar content is desirable to serve as a substrate for color formation.

The pH at the end of fermentation (Table 2) was in the range of 3.61–3.94, which is within the expected range for sourdough [21]. A longer fermentation time is usually applied to bran [35] and consequently provided a lower pH (Table 2), possibly influencing the decrease in yeast CFU/g [18]. As expected, pH was higher in the fermentations that ended with a lower LAB CFU/g count and was more significantly affected by L-LA concentration (R^2^ = 0.71), the dominant organic acid in all fermentations. Sugar analysis was used to determine in which fermentations the sugar content of the raw materials was sufficient to support fermentation to completion. The interactions (Table 3) between PMF and CSPF as well as PMB and CSPF had a significant influence on the number of LAB CFU/g as well as all other parameters, indicating a complementary effect of CSPF and PMF in sourdough ferments. Both LF and KM are able to metabolize sucrose, glucose, and fructose, and no correlation was found between the remaining sugar concentration at the end of fermentation and the cell density of LAB or yeast. CSPF was the dominant source of sugar in the fermentations, since all samples with CSPF contained sugar at the end of fermentation, while the sourdoughs without CSPF (runs 1, 4, 10, and 11) contained no sugar at the end of fermentation (Table 2), indicating the possibility that the sugar content was not sufficient to support the growth of lactobacilli and yeast to full potential in these fermentations.

**Figure 1:**
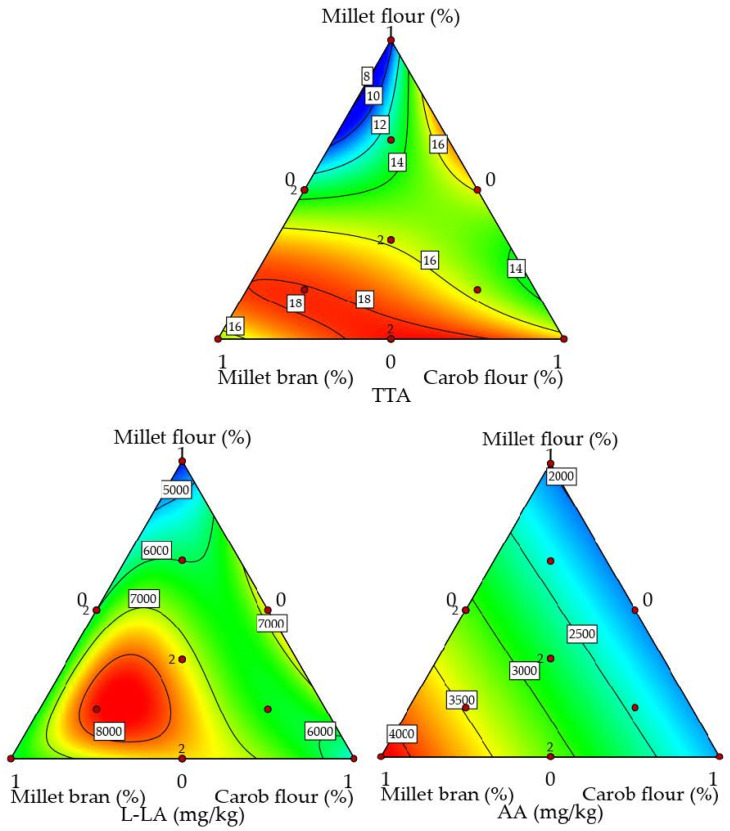
Response surface plots of the effects of proso millet flour (PMF), proso millet bran (PMB), and carob seed and pod flour (CSPF) on total titratable acidity (TTA) and amount of L-lactic acid (L-LA) (mg/kg) and acetic acid (AA) (mg/kg). Red color indicates the highest impact, while the blue indicates the weakest.

A fermentation temperature of 36 °C that is optimal for *L. fermentum* [18] favored the growth of the thermophilic yeast *K. marxianus* and enabled rapid proliferation of LAB and thus the production of organic acids [21]. The amount of AA was between 1718 and 4108 mg/kg sourdough, while the amount of L-LA was between 4225 and 8397 mg/kg sourdough. The AA concentration did not correlate with the LAB or yeast CFU/g. The lower concentration of the AA compared to L-LA is due to the fact that AA production is primarily influenced by the starter culture and aeration conditions, and *L. fermentum* primarily produces lactic acid [36]. Additionally, the fermentations were carried out in a sealed closed jar, leading to limited aeration. The use of millet bran favored the production of AA (R^2^ = 0.947) (Figure 1). The highest amount of AA was found in the sourdough made from PMB only, followed by the sourdough made from a flour blend with the highest PMB content (3641 mg/kg). The lowest AA and L-LA concentrations were determined in the single fermentation of PMF. Decortication that is applied to proso millet [37] results in a loss of fiber and mineral content, which provide a substrate for microbial activity [38]. The L-LA concentration showed a positive correlation with the number of LAB CFU/g (R^2^ = 0.706). On the other hand, a negative correlation was found between the L-LA concentration and the number of yeast CFU/g (R^2^ = −0.62). Due to its higher concentration, L-LA had a stronger inhibitory effect on the yeast count. The L-LA concentration was strongly influenced by the interaction of PMB and CSPF as well as PMF and CSPF (Table 3).

The values for TTA 9.5–18.4 M 0.1 NaOH/10 g dough corresponded to the range expected in the literature [21]. A higher amount of PMF lowered the TTA (R^2^ = −0.870) of the sourdough. The TTA was highest in fermentations with PMB and CSPF. The higher mineral content of these flours probably influenced the buffering capacity of the sourdough system, resulting in a higher TTA [39]. The TTA was also strongly influenced by the L-LA content (R^2^ = 0.81), but not by the AA content (R^2^ = 0.34), as the concentration of L-LA in the sourdoughs was on average more than twice as high as the concentration of AA. This higher concentration of L-LA, notable from the FQ (Table 2), contributes more significantly to the overall acidity. Since it is more prone to dissociation, lactic acid primarily affects acidification [40]. In addition, the dough yield (DY) was 400 in all fermentations, which is a high DY according to the literature [41]. A higher DY is expected to promote lactic acid production compared to acetic acid, as the yeast is not inhibited by the acetic acid concentration [18].

Although a possible fermentation quotient (FQ) of up to 20 is given in the literature [21], it is assumed that it should optimally be below 5, which was the case in all our fermentations. The FQ was lowest in the single fermentations of CSPF, PMB, and PMF, respectively (Table 2), while it was highest in the combined fermentation of PMF and CF, followed by the fermentation of all three flours in the same amount. Numerical optimization offered fifteen solutions, all containing a mixture of all three flours. The first solution had a desirability value of 0.729 and contained all three flours in a PMB/PMF/CSPF ratio of 2.4:1:1.2. The optimal solution was consistent with the noted positive effects of combining all three flours with a larger amount of PMB observed for the individual reaction variables.

### 3.2. Optimized Fermentation Performance of LF + KM Compared with a Commercial Starter LV1

**Figure 2:**
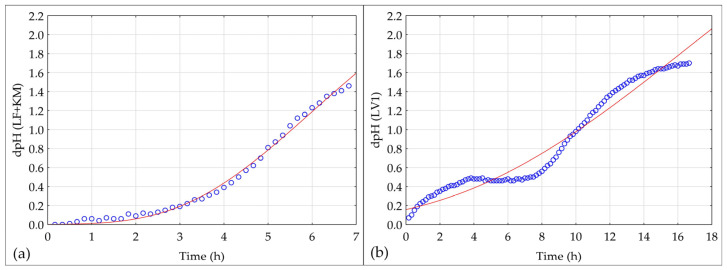
Acidification kinetics fitted to the Gompertz model for sourdough fermented at 35 °C with (**a**) *Limosilactobacillus fermentum* and *Kluyveromyces marxianus* (LF + KM), and with (**b**) starter LV1, for 7 and 16 h, respectively. Circles represent the measured ΔpH values at different time points. The solid line illustrates the fitted Gompertz model, which describes the acidification process as a function of time.

The optimum flour blend ratio for fermentation with LF + KM was also determined by comparing the acidification kinetics of the two with fermentation of the same blend with a commercial starter LV1. Although the fermentations were carried out under the same conditions and with the same medium, with a high DY to support the acidification kinetics, significant differences were observed between the fermentations. The fermentations were left until a pH of 4.1 was reached, which took 6.5 h for LF + KM and 16 h for LV1 (Figure 2, Table 4). A starter culture for a semi-liquid fermented dough ideally consists of LAB species that acidify the mixture quickly [18]. The curves show a typical sigmoidal shape characteristic of the acidification kinetics (Figure 2) [42], while the *p*-values and R^2^ for both fermentations indicate a good fit to the Gompertz model. LF + KM takes about one hour less to start acidification (Table 4), while the maximum acidification rate of LF + KM is 2.6 higher than that of LV1 (Table 4), indicating a faster acid production of LF + KM. Apart from the rate of acidification, the fermentations also differed significantly in the duration of the lag phase and its onset. LV1 had a much longer lag phase, indicating a slower initial adaptation and metabolic activity of the microorganisms. It is possible that the combination of *L. fermentum* and *K. marxianus* leads to synergistic interactions that improve acidification kinetics. LF + KM has a shorter duration of the exponential phase compared to LV1 due to a faster reach of the maximum. The final success of the fermentations was comparable, with the amount of L-LA in the optimized LF + KM sourdough being 5870 mg/kg sourdough, while in the LV1 sourdough, it was 6800 mg/kg sourdough. A faster acidification rate of LF + KM led to higher production of acetic acid (2680 mg/kg sourdough) than that of LV1 sourdough (2380 mg/kg sourdough), resulting in a higher TTA for the LF + KM sourdough (12.98) than for the LV1 sourdough (9.8). Nevertheless, the LF + KM resulted in a satisfactory FQ of 1.49, while the LV1 sourdough had an FQ of 1.94.

### 3.3. Impact of Sourdoughs on the Quality of Gluten-Free Bread

The efficiencies of the LF + KM and LV1 fermentation were further compared by evaluating the quality of the GFFB with sourdough addition.

The measured hardness and volume of the whole GFFB were comparable to the crumb hardness measured in our previous study with proso millet, pumpkin seed cake, and buckwheat flour [12], which also indicates a soft crust of the GFFB. Bread with sourdough fermented with LF + KM (CSPF_LF + KM) showed an increase in volume by 26% compared to bread with LV1 sourdough (CSPF_LV1), which in turn was reflected in a decrease in hardness and chewiness by 31% and 27%, respectively, and an improvement in the shape of the GFFB by 17%. The study of Matos and Rosell also observed similar traits for chewiness and hardness in gluten-free breads. By their definition, a decrease in chewiness indicates the bread breaks more easily in the mouth, a trait that is desirable for gluten-free breads that usually present a high hardness [43] This confirms previous studies that have shown that *Limosilactobacillus fermentum* strains isolated from quinoa and buckwheat improve the technological properties of bread, including a higher specific volume and a softer crumb [44]. Since gluten-free breads exhibit reduced cohesiveness and resilience [45,46], the statistically significant improvement of cohesiveness of the CSPF_LF + KM (Table 5) is of interest for the GF FB due to its specific properties. Cohesiveness represents the extent to which a material can be deformed, or amount of sample deformation when biting, before it ruptures [43], and GFFB, is in average 1 cm in height, and 13 cm in diameter, and therefore shows a larger possibility of fractures in the middle of the bread than regular gluten-free breads. The difference in the resilience—a trait that can indicate the elasticity of the bread [47]—of the samples was not statistically significant. Although hydration properties show positive correlations with cohesiveness and resilience [43], there was no significant difference in the baking loss of two samples. Gluten-free bread crust color is an important sensory attribute that has a significant effect on consumer acceptance. Since the breads were baked in the same conditions and with the same ingredients, the fermentation process itself could be responsible for the differences in color. The differences in sugar content at end of fermentation due to use of different starter cultures could lead to differences in color [39]. The CSPF_LV1 bread had a higher b* value (3.3) and a lower a* value (0.9) than the CSPF_LF + KM bread (b* = 2.6; a* = 1.2), resulting in a higher browning index (Table 5). Considering that generally, gluten-free breads have a pale crust color [48], the high browning index of both breads is satisfactory. A higher browning index of CSPF_LV1 is consistent with the slightly darker crustfound in CSPF_LF + KM (52.3), than the one in CSPF_LV1 (53.3). Both breads showed satisfactory physical properties, a confirmation of the potential that sourdough has in gluten-free bread production, with the chosen starter cultures having a more efficient effect.

## 4. Conclusions

Proso millet flour, proso millet bran, and carob flour showed good fermentation performance using *L. fermentum* with *K. marxianus* and could therefore be successfully used for the production of sourdough for gluten-free flatbreads. In particular, the raw materials used showed complementary effects on LAB and yeast CFU/g, pH, TTA, and the production of L-lactic and acetic acid with the optimized ratio of 2.4:1:1.2 (PMB:PMF:CSPF) for proso millet flour, proso millet bran, and carob flour, respectively. The starter culture used showed better fermentation performance compared to commercial starter cultures. The combination of *L. fermentum* and *K. marxianus* proved to be efficient in improving acidification kinetics and achieving a desirable fermentation quotient. The use of this sourdough improved the softness, elasticity, chewiness, shape, and volume of the gluten-free flatbread. The research results emphasize the success of using sourdough in the production of gluten-free bread. Further research should investigate the application of selected starter cultures on sourdough production of other gluten-free alternative ingredients, as well as the effect of sourdough on dough rheological properties.

## Figures and Tables

**Table 1:** Experimental design used for evaluation of the fermentation performance of proso millet flour and bran and carob seed and pod flour.

Run	1	2	3	4	5	6	7	8	9	10	11	12	13
PMF%	100	0	0	50	33.3	16.7	16.7	0	33.3	50	0	50	66.7
PMB%	0	50	0	50	33.3	66.7	16.7	50	33.3	50	100	0	16.7
CSPF%	0	50	100	0	33.3	16.7	66.7	50	33.3	0	0	50	16.7

Abbreviations: PMF—proso millet flour; PMB—proso millet bran; CSPF—carob seed and pod flour.

**Table 2:** Variables used in assessment of fermentation performance of carob flour, proso millet flour, and proso millet bran measured at the end of 16 h fermentations.

Run	LAB (CFU/g)	Yeast (CFU/g)	pH	Fructose	Glucose	Saccharose	FQ
				(mg/g Sourdough)			
1	3.9 × 10^7^	7.2 × 10^6^	3.81	n.d.	n.d.	n.d.	1.68 ^d^
2	6.4 × 10^8^	12.7 × 10^5^	3.76	1.90	0.90	n.d.	1.71 ^d^
3	2.8 × 10^8^	1.3 × 10^6^	3.94	2.80	0.80	n.d.	1.74 ^d^
4	8.2 × 10^8^	3.5 × 10^5^	3.77	n.d.	n.d.	n.d.	1.21 ^b^
5	1.6 × 10^9^	4.2 × 10^5^	3.68	0.83	0.83	n.d.	1.92 ^e^
6	1.2 × 10^9^	8.0 × 10^5^	3.62	0.40	0.30	n.d.	1.56 ^c^
7	5.4 × 10^8^	2.5 × 10^5^	3.76	1.60	1.55	0.23	1.60 ^cd^
8	7.8 × 10^8^	1.7 × 10^5^	3.72	1.90	0.90	n.d.	1.71 ^d^
9	1.8 × 10^9^	7.0 × 10^5^	3.64	0.83	0.85	n.d.	1.92 ^e^
10	8.6 × 10^8^	4.2 × 10^5^	3.75	n.d.	n.d.	n.d.	1.21 ^b^
11	5.6 × 10^8^	3.4 × 10^6^	3.86	n.d.	n.d.	n.d.	1.00 ^a^
12	9.3 × 10^8^	8.0 × 10^5^	3.61	1.25	0.68	0.53	2.14 ^f^
13	7.6 × 10^8^	1.8 × 10^6^	3.63	0.40	0.60	n.d.	1.84 ^e^

Abbreviations: LAB—lactic acid bacteria; CFU—colony-forming units; FQ—fermentation quotient; n.d.—not detected (<0.10 mg/g). Values within the same column marked with different letters differ significantly according to Tukey’s test (*p* < 0.05).

**Table 3:** Analysis of variance and lack of fit test results generated by RSM for all response variables.

	TTA	L-LA	AA	LAB	YEAST	pH
Intercept	15.291	6556	2814	8.27 × 10^8^	23.83 × 10^5^	3.735
Model *p* value	<0.0001	<0.0001	<0.0001	0.0047	0.0015	0.0065
PMF × PMB						
F value	23.75	171.58	-	44.96	43.79	-
*p* value	0.0082	0.0002	-	0.0068	0.0027	-
PMF × CSPF						
F value	156.92	1094.31	-	60.02	111.25	20.91
*p* value	0.0002	<0.0001	-	0.0045	0.0005	0.0018
PMB × CSPF						
F value	155.95	762.07	-	13.28	107.26	9.21
*p* value	0.0002	<0.0001	-	0.0356	0.0005	0.0162
PMF2 × PMB × CSPF						
F value	19.63	48.70	-	74.92	8.93	-
*p* value	0.0114	0.0022	-	0.0032	0.0404	-
PMF × PMB2 × CSPF						
F value	-	490.80	-	-	-	-
*p* value	-	<0.0001	-	-	-	-
PMF × PMB × CSPF2						
F value	-	299.47	-	-	-	-
*p* value	-	<0.0001	-	-	-	-
PMF × PMB (PMF-PMB)						
F value	118.23	-	-	60.87	8.09	-
*p* value	0.0004	-	-	0.0044	0.0467	-
PMF × CSPF (PMF-CSPF)						
F value	129.39	-	-	23.81	8.91	-
*p* value	0.0004	-	-	0.0165	0.0405	-
PMF × PMB (PMF-PMB)2						
F value	-	-	-	35.60		-
*p* value	-	-	-	0.0094		-
R^2^	0.998	0.999	0.899	0.993	0.987	0.802
Adjusted R^2^	0.994	0.997	0.879	0.971	0.962	0.703
Predicted R^2^	0.866	0.413	0.822	-	−3.7460	−0.469
Lack of fit						
F value	0.413	-	-	-	7.28	7.19
*p* value	0.566	-	-	-	0.074	0.07

Abbreviations: RSM—response surface methodology; TTA—total titratable acidity; L-LA—l-lactic acid; AA—acetic acid; PMF—proso millet flour; PMB—proso millet bran; CSPF—carob seed and pod flour.

**Table 4:** Acidification kinetics according to the Gompertz model for sourdough fermented with *Limosilactobacillus fermentum* and *Kluyveromyces marxianus* (LF + KM), and with a commercial starter Livendo^®^LV1 (LV1).

	μmax (h-1)	A (dpH)	Λ (h)	Ti (h)	R^2^
LF + KM	0.40	2.91	2.69	5.38	0.9971
LV1	0.15	5.47	3.72	17.51	0.9800

Abbreviations: μmax (h-1)—maximum acidification rate; A (dpH)—difference in pH (units) between the initial value and the value reached in the stationary phase of the sourdough fermentation; Λ (h)—lag phase duration; Ti (h)—time to reach μmax; R^2^—coefficient of determination.

**Table 5:** Effect of addition of sourdough fermented with *Limosilactobacillus fermentum* and *Kluyveromyces marxianus* (CSPF_LF + KM) or sourdough fermented with starter Livendo^®^LV1 (CSPF_LV1) on physical properties of gluten-free flatbread.

	CSPF_LV1	CSPF_LF + KM
	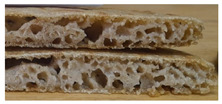	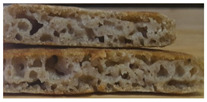
Hardness (N)	77.4 ± 2.6 ^a^	52.8 ± 3.5 ^b^
Cohesiveness	0.53 ± 0.01 ^a^	0.58 ± 0.02 ^b^
Chewiness (Nmm)	257 ± 12 ^a^	186 ± 13.5 ^b^
Resilience	0.45 ± 0.01 ^a^	0.48 ± 0.02 ^a^
Baking loss%	20.6 ± 0.3 ^a^	20.9 ± 0.9 ^a^
Specific volume (mL/g)	1.48 ± 0.05 ^a^	2.0 ± 0.05 ^b^
Shape (d/h)	11.4 ± 0.7 ^a^	13.3 ± 0.8 ^b^
Browning index	7.54	6.73

Abbreviations: CSPF-carob seed and pod flour. Mean ± standard deviation values are shown for each row. Values within the same row marked with different letters differ significantly according to Tukey’s test (*p* < 0.05). The measurements were done at minimum of 6 replications for texture properties and a minimum of 3 replications for others.

## Data Availability

The original contributions presented in the study are included in the article, further inquiries can be directed to the corresponding author.
